# Supplementary material for: Sleep-like cortical OFF-periods disrupt causality and complexity in the brain of unresponsive wakefulness syndrome patients
Source: Nat Commun. 2018 Oct 24;9:4427. doi: 10.1038/s41467-018-06871-1 (PMC6200777; doi:10.1038/s41467-018-06871-1)
Supplement: Supplementary file 1 — Supplementary Information [file 41467_2018_6871_MOESM1_ESM.pdf]

Supplementary Information of

Sleep-like cortical OFF-periods disrupt causality and complexity in the brain of Unresponsive Wakefulness Syndrome patients

Rosanova, Fecchio et al.

Supplementary Methods

TMS equipment

Stimulation pulses were delivered with a Focal Bipulse figure-of-eight coil (mean/outer winding diameter ~50/70 mm, biphasic pulse shape, pulse length ~280 μs, focal area of the stimulation 0.68 cm^2) driven by a Mobile Stimulator Unit (eXimia TMS Stimulator, Nexstim Ltd., Finland).

EEG recordings

EEG data were recorded using a TMS-compatible 60-channel amplifier (Nexstim Ltd, Finland), which gates the magnetic pulse artefact and provides artifact-free data from 8 ms after stimulation [1]. Raw recordings were referenced to a forehead electrode, online filtered between 0.1-350 Hz, and sampled at 1450 Hz. Two additional sensors were applied to record the electrooculogram (EOG). As previously recommended [2], during all TMS/EEG recordings a masking sound was played via earphones and a thin layer of foam was placed between the coil and the scalp in order to abolish the auditory potentials evoked by the TMS loud click.

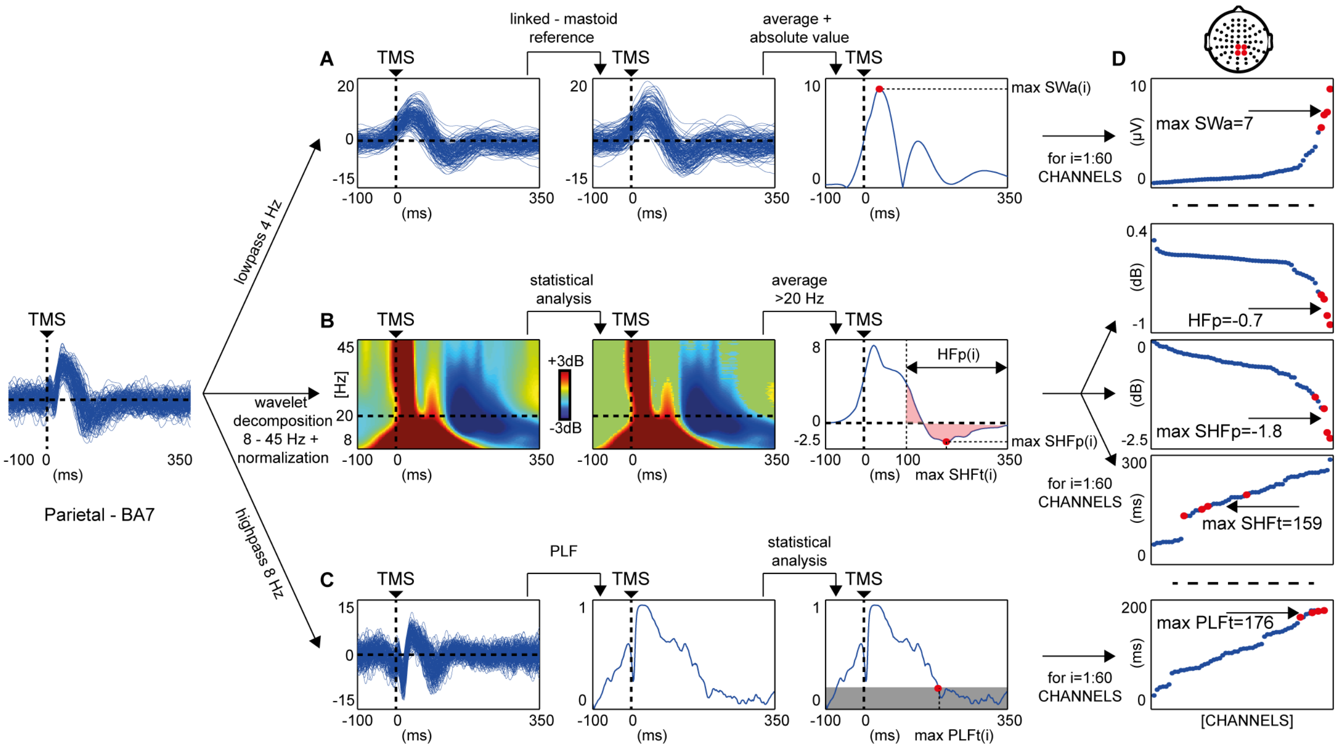

Supplementary Figure 1. Data analysis procedure. In order to assess (1) the occurrence of a TMS-evoked slow wave (2) associated with the presence of a cortical OFF-period and (3) the impact of the OFF-periods on local causal interactions, we measured (1) the amplitude of the low frequency (< 4 Hz) components, (2) the significant high frequency (> 20 Hz) suppression of EEG power compared to baseline and (3) the broadband (> 8 Hz) phase-locking factor (PLF). Specifically, for each EEG channel *i* (from 1 to 60), we performed three different procedures starting from single TMS/EEG trials that are represented on the left (thin blue traces). **(A)** For calculating the amplitude of low-frequency components we first low-pass filtered single trials below 4 Hz (thin blue traces, left panel) using a third order Chebyshev filter, then re-referenced the data to the mathematically linked mastoids (thin blue traces, middle panel) and calculated the absolute value of the average of filtered trials (thick blue traces, right panel). Finally, we

calculated the amplitude of low frequency components as the maximum of the signal between 8 and 350 ms (SWa_i, red dot in the plot on the right). **(B)** For calculating the high frequency (> 20 Hz) suppression of EEG power we used the *newtimef* EEGLAB routine [3]. Specifically, the ERSP of each channel *i* was obtained by decomposing each single trial in the time-frequency domain by a means of a Wavelet transform (Morlet, window span: 3.5 cycles) using a sliding window [4]. Each ERSP was then normalized performing the *full-epoch length single-trial correction*, averaged across trials and baseline corrected (between -350 and -100 ms before the TMS pulse, left panel) [3,5]. Non-significant activity was set to zero using a bootstrap statistics ($\alpha < 0.05$, number of permutations = 500) with respect to baseline and colored in green (middle panel). Finally, ERSP values above 20 Hz were averaged (thick blue trace, right panel) to calculate the amount of EEG high frequency power (HFp_i) as the average between 100 and 350 ms (shaded red area), as well as the extent (max $SHFp_i$) and timing (max $SHFt_i$) of the maximum EEG high frequency suppression, as indicated by the red dot. **(C)** To calculate PLF, single trials were first high pass filtered above 8 Hz (thin blue traces, left panel) using a third order Butterworth filter. Then, PLF was calculated as the absolute value of the average of the Hilbert Transform across all trials (middle panel). Statistically significant differences from baseline (between -500 ms and -100 ms) were assessed assuming a Rayleigh distribution of the baseline values. Finally, PLF values below a given statistical threshold ($\alpha < 0.01$, shaded gray area) were set to zero (right panel) and the last significant time point was considered (max $PLFt_i$, red dot). **(D)** For each measure described above we calculated the average (black arrows) of the four channels (red dots on the EEG cap layout) closest to the stimulation site (here, BA7).

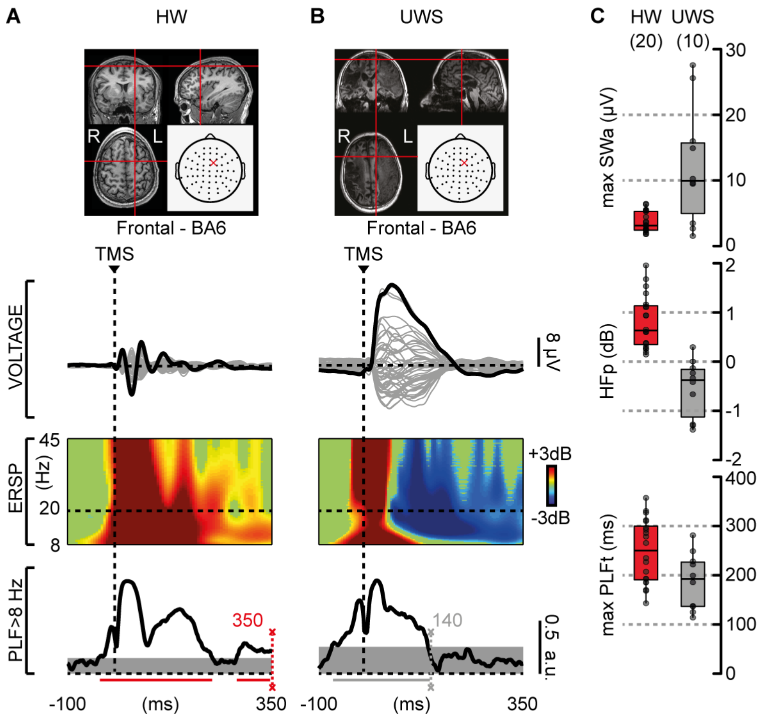

Supplementary Figure 2. TMS-evoked responses of the frontal cortex, ERSP and PLF in awake healthy subjects and UWS patients. Results for a representative healthy subject during wakefulness (HW) and a representative UWS patient (patient 12 in supplementary Table 2) are shown. **(A and B)** MRIs and cortical targets as estimated by Navigated Brain Stimulation system are shown (top row). A dashed vertical line marks the occurrence of TMS. The butterfly plots of TMS-evoked EEG potentials recorded at all 60 channels (gray traces) are depicted. ERSP and PLF are presented for the electrode with the largest response (black trace). In the ERSP plot, the dashed horizontal line indicates the 20 Hz frequency bin. The colored horizontal line at the bottom indicates PLF time points above statistical threshold (shaded gray area). The colored-dashed vertical line indicates the timing of the last significant ($\alpha < 0.01$) PLF time point. **(C)** From top to bottom, boxplot of slow wave amplitude (max SWa < 4 Hz), high frequency power (HFp)

and duration of PLF (max PLFt) for awake healthy subjects (red) and UWS patients (gray) are shown. Boxplot displays the median (center line), the first and third quartiles (bounds of box). The whiskers extend from the bound of the box to the largest/smallest value no further than 1.5* inter-quartile range. Outlier datapoints are indicated by dots outside whiskers. Statistical comparison between groups are reported in Supplementary Table 1

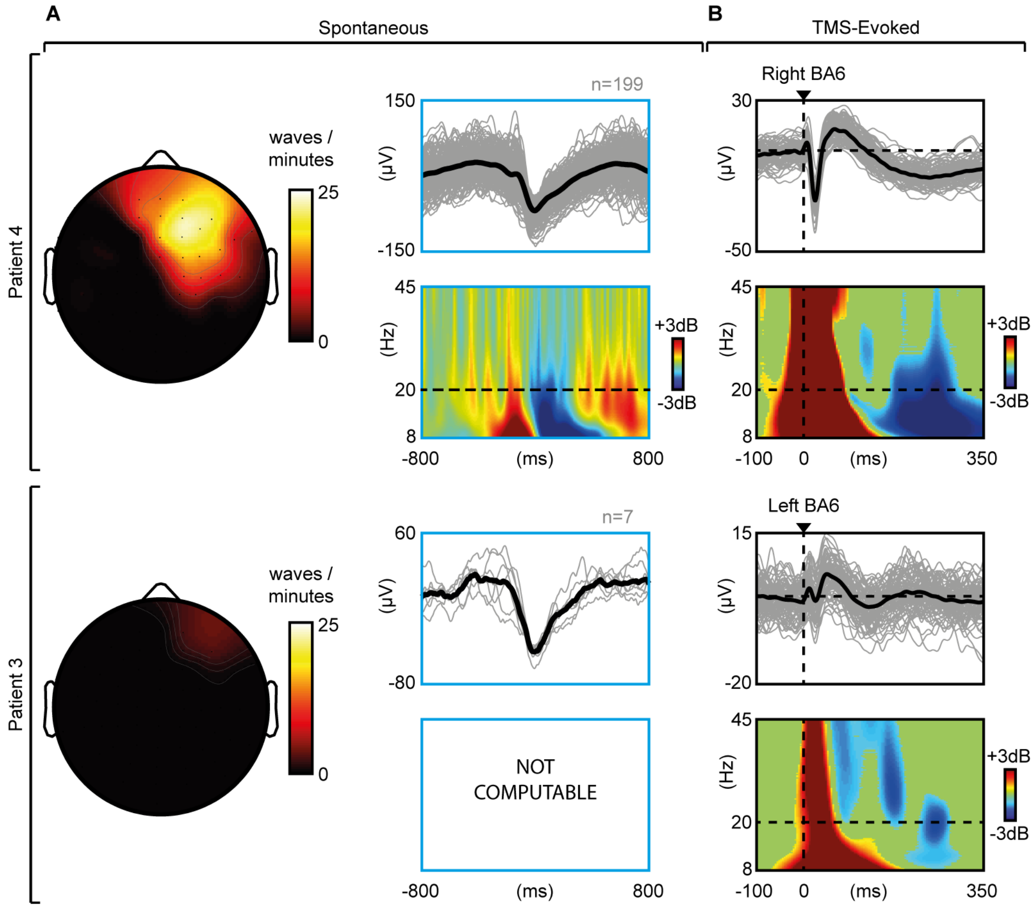

Supplementary Figure 3. EEG recordings performed in awake UWS patients display a highly variable prevalence of spontaneous sleep-like slow waves and OFF-periods, which are invariably revealed by the TMS. In two representative UWS patients (patient 3 and 4) the

prevalence of slow-wave activity was assessed in the background EEG according to a well-established automatic detection algorithm [6]. **(A)** For both patients, on the left the topographical distribution of spontaneous slow wave density is shown. Grey traces in the cyan boxes represent individual spontaneous slow waves in the channel showing the maximum number of detections (waves/min), together with their average (black line), and the corresponding ERSP (bottom panel) **(B)** For both patients, single trials (grey traces) of the electrode showing the largest TMS-evoked potential (average responses are superimposed in black), together with the corresponding ERSP (bottom panel). Dashed vertical lines mark the occurrence of TMS. In the ERSP plots, the dashed horizontal lines indicate the 20 Hz frequency bin. This figure shows that i) slow waves (and the associated OFF-periods) can be immediately evident from the spontaneous EEG, and ii) TMS perturbations reveal the presence of OFF-periods even in those patients in whom slow waves are not prevalent.

	**Wilcoxon ranksum test (P) HW (N = 20) vs UWS (N = 10)**
**max Swa**	0.010
**HFp**	$2.652*10^{-5}$
**max PLFt**	0.031

Supplementary Table 1. Statistical analyses between groups stimulated over BA6. Statistical comparison regarding boxplots of slow wave amplitude (max SWa), high frequency power (HFp), and duration of PLF (max PLFt) presented in Supplementary Fig. 2C. Specifically,

details regarding the applied tests, the sample size and the significance values for each comparison between conditions (HW, UWS) are reported.

Clinical features					CRS-R								Rest EEG	TMS-EEG	
Patient		Gender (Age)	Etiology	S/C condition	Diagnosis at time of TMS/EEG	Au	Vis	Mot	O/V	Comm	Ar	Total score	EEG category	Targeted area	PCImax
**1**		M (81)	A	S	VS/UWS	0	0	2	1	0	1	4	SE	BA6R/BA7L	0.20
**2**		M (68)	T	S	VS/UWS	0	0	2	0	0	2	4	SE	BA7L	0.20
**3**		F (83)	T	S	VS/UWS	1	0	1	0	0	1	3	SE	BA6L/BA7L	0.30
**4**		M (19)	T	C	VS/UWS	1	0	1	1	0	1	4	MO	BA6R/BA7R	0.29
**5**		F (77)	V	S	VS/UWS	1	0	2	1	0	1	5	SE	BA7L	0.21
**6**		M (67)	V	C	VS/UWS	2	0	1	1	0	2	6	MO	BA7L	0.28
**7**		M (34)	A	C	VS/UWS	1	1	2	1	0	2	7	SE	BA6L/BA7R	0.29
**8**		F (19)	T	C	VS/UWS	1	1	1	2	0	1	6	MO	BA6R	0.13
**9**		M (55)	A	S	VS/UWS	2	1	0	0	0	2	5	SE	BA6L	0.23
**10**		M (57)	A	C	VS/UWS	1	0	2	1	0	2	6	SE	BA7R	0.22
**11**		F (44)	A	C	VS/UWS	1	0	2	2	0	2	7	MO	BA6R/BA7R	0.24
**12**		F (60)	A	S	VS/UWS	0	0	1	1	0	1	3	MO	BA6R/BA7L	0.20
**13**		M(57)	A	C	VS/UWS	1	0	2	1	0	1	5	MO	BA7R	0.29
**14**		M(56)	T	C	VS/UWS	1	0	1	1	0	1	4	MO	BA7R	0.21
**15**		M(57)	V	S	VS/UWS	0	0	1	1	0	1	3	MO	BA6R/BA7R	0.23
**16**	1	F(60)	A	S	VS/UWS	0	0	2	2	0	1	5	SE	BA6R/BA7R	0.25
	2				MCS	3	0	2	1	1	1	8	SE	BA7L	0.33
	3				EMCS	4	5	6	2	2	1	20	MI	BA7L	0.40

Supplementary Table 2. Clinical and electrophysiological data. VS/UWS = vegetative state/unresponsive wakefulness syndrome, MCS = minimally conscious state, EMCS = emergence of the minimally conscious state; S = sub-acute, C = chronic; CRS-R = Coma Recovery Scale-Revised, A = anoxic, T = traumatic, V = vascular accident; SE = severe, MO = moderate; MI = mild as per [7]; BA = Brodmann Area. Note that CRS-R sub-scores (Au = auditory, Vis = visual, Mot =

motor, O/V = Oromotor/Verbal function, Comm = communication, Ar = arousal) are relative to the day when the TMS/EEG session was performed. PCImax indicates the maximum PCI value across sessions.

Subject	Gender (Age)	TMS-EEG during Wakefulness		TMS-EEG during Sleep	
		Targeted area	PCImax	Targeted area	PCImax
**1**	F (25)	BA6L/BA7L	0.48	BA7L	0.14
**2**	M (30)	BA6R/BA7R	0.64	BA7R	0.30
**3**	M (49)	BA6R/BA7R	0.55	BA7R	0.26
**4**	M (38)	BA6L/BA7L	0.48	BA7L	0.25
**5**	F (21)	BA6R/BA7R	0.46	BA7R	0.29
**6**	F (31)	BA6L/BA7L	0.52	BA7L	0.25
**7**	M (21)	BA6L/BA7L	0.44	BA7L	0.14
**8**	F (24)	BA6R/BA7R	0.55	BA7R	0.12
**9**	M (28)	BA6L/BA7L	0.57		
**10**	M (75)	BA6L/BA7L	0.50		
**11**	F (26)	BA6R/BA7R	0.56		
**12**	F (39)	BA6R/BA7R	0.51		
**13**	M (19)	BA6R/BA7R	0.45		
**14**	M (45)	BA6L/BA7L	0.64		
**15**	M (40)	BA6R/BA7R	0.57		
**16**	F (60)	BA6L/BA7L	0.63		
**17**	M (35)	BA6R/BA7R	0.64		
**18**	M (80)	BA6L/BA7L	0.57		
**19**	F (33)	BA6R/BA7R	0.55		
**20**	M (58)	BA6L/BA7L	0.51		

Supplementary Table 3. Demographics, TMS-EEG stimulation site and PCI value for healthy participants. BA = Brodmann Area.

Supplementary References

1. Virtanen, J., Ruohonen, J., Näätänen, R. & Ilmoniemi, R. J. Instrumentation for the measurement of electric brain responses to transcranial magnetic stimulation. *Med. Biol. Eng. Comput.* **37,** 322–326 (1999).
2. ter Braack, E. M., de Vos, C. C. & van Putten, M. J. A. M. Masking the Auditory Evoked Potential in TMS-EEG: A Comparison of Various Methods. *Brain Topogr.* **28,** 520–528 (2015).
3. Grandchamp, R. & Delorme, A. Single-Trial Normalization for Event-Related Spectral Decomposition Reduces Sensitivity to Noisy Trials. *Front Psychol* **2,** 236 (2011).
4. Rosanova, M. *et al.* Natural frequencies of human corticothalamic circuits. *J. Neurosci.* **29,** 7679–7685 (2009).
5. Fecchio, M. *et al.* The spectral features of EEG responses to transcranial magnetic stimulation of the primary motor cortex depend on the amplitude of the motor evoked potentials. *PLoS One* **12,** e0184910 (2017).
6. Riedner, B. A. *et al.* Sleep homeostasis and cortical synchronization: III. A high-density EEG study of sleep slow waves in humans. *Sleep* **30,** 1643–1657 (2007).
7. Forgacs, P. B. *et al.* Preservation of electroencephalographic organization in patients with impaired consciousness and imaging-based evidence of command-following. *Ann. Neurol.* **76,** 869–879 (2014).
